# “… because I'm so drunk at the time, the last thing I'm going to think about is calories”: Strengthening the argument for Drunkorexia as a food and alcohol disturbance, evidence from a qualitative study

**DOI:** 10.1111/bjhp.12594

**Published:** 2022-04-06

**Authors:** Katharina Sophie Vogt, Michela Harper, Bethany Leigh Griffin

**Affiliations:** ^1^ Bradford Institute for Health Research Bradford Royal Infirmary UK; ^2^ School of Psychology University of Leeds UK; ^3^ Department of Psychology The University of Huddersfield, Queensgate UK; ^4^ Department of Psychology University of Sheffield UK

**Keywords:** alcohol, appearance, binge‐drinking, compensatory behaviours, Drunkorexia

## Abstract

**Objectives:**

Drunkorexia are inappropriate compensatory behaviours in response to alcohol consumption (restricting food intake, excessive exercise, and purging). Past (predominantly quantitative) research shows that Drunkorexia is prevalent in 18–26‐year‐olds, and has significant negative health‐related consequences. There is a debate whether Drunkorexia constitutes an eating or substance disorder, or a Food and Alcohol Disturbance (FAD). To further explore this, and understand underlying motivators, this study utilized qualitative methods.

**Design:**

Qualitative interviews with ten participants (aged 18–26).

**Methods:**

Interviews were analysed with Thematic Analysis.

**Results:**

Three themes were developed: (1) Appearance concerns as motivators, (2) Drunkorexia behaviours to get value for money, and (3) “It’s just a pattern… something I’ve always done”: Drunkorexia as a routine. Results show that Drunkorexia is driven by appearance‐related concerns, such as, wanting to look better/slimmer, engaged in, in relation to an event, such as going out drinking, and carried out despite negative health‐related consequences. However, disregard for compensatory behaviours once drunk was also described, culminating in the consumption of high‐calorie food. This suggests that Drunkorexia is not a persistent pattern of maladaptive behaviour as found in eating or substance use disorders. Wanting value for money (i.e., feeling the maximum intoxication) was described as another reason for Drunkorexia engagement; thus showing that participants consider compensatory behaviours part of their routine of going out drinking.

**Conclusions:**

These result support the view of Drunkorexia as a FAD, rather than an eating or substance use disorder, and show that 18–26‐year‐olds are an at‐risk group for Drunkorexia and its negative health‐related consequences.

**Statement of contribution**

***What is already known on the subject?***

Drunkorexia behaviours are inappropriate compensatory behaviours in response to alcohol consumption, such as restricting food intake, purging, and use of laxatives. Originally, it was thought that Drunkorexia was only associated with university students. However, recent research has confirmed that this behaviour is prevalent across 18–26‐year‐olds. To date, Drunkorexia literature is dominated by quantitative research, assessing potential predictors. A broad variety of predictors have been proposed, ranging from weight esteem to increased disinhibition.

***What does this study add?***

- Drunkorexia engagement is a routine for participants, predominantly motivated by appearance‐related concerns, such as not wanting to look bloated on nights out drinking or on social media photos, and wanting ‘value for money’ for alcohol purchased.
- However, participants also reported disregard for Drunkorexia behaviours for appearance‐related motivators at the end of drinking events, characterized by consumption of high‐calorie food.
- Thus, this study adds evidence that Drunkorexia be classified as a Food and Alcohol Disturbance (FAD), rather than a continuous, maladaptive disorder, and highlights the prevalence of FAD in 18–26 year‐olds.

## Background

Drunkorexia is a term first coined by the media to refer to inappropriate compensatory behaviours that are carried out in response to alcohol consumption (Kershaw, 2008). While initially thought to be motivated by weight maintenance, it is now thought to be a complex behaviour with multiple motivators and predictors, such as weight esteem, appearance esteem, and sensation‐seeking (Griffin & Vogt, 2020; Hill & Lego, 2019; Hunt & Forbush, 2016). Compensatory behaviours include restricting food intake, reducing the intake of high‐calorie and fatty foods, excessive exercise, and purging (Booker, Novik, Galloway, & Holmes, 2021; Laghi, Pompili, Bianchi, Lonigro, & Baiocco, 2019; Pompili & Laghi, 2020).

In Laghi et al. (2019)’s exploratory study, it was found that 23% of their student sample engaged in Drunkorexia behaviours. Knight, Castelnuovo, Pietrabissa, Manzoni, and Simpson (2017) reported that 57.7% of their Australian student sample reported engaging in one or more Drunkorexia behaviours (purging; self‐induced vomiting, the use of laxatives and/ or diuretics, restricting calories, and excessive exercise) at least 25% of the time when alcohol use was intended. Despite highlighting the prevalence of these behaviours, it has to be noted that both studies, and the majority of the literature, uses student samples (Bryant, Darkes, & Rahal, 2012; Kelly‐Weeder, 2011; Roosen & Mills, 2015), perhaps for convenience. This has led to the mis‐conception that these Drunkorexia behaviours are a student lifestyle choice associated with university culture, similar to binge‐drinking (Barry & Piazza‐Gardner, 2012; Wilkinson & Ivsins, 2017). There is now however increasing evidence to suggest that the behaviours are prevalent outside of the student population (Griffin & Vogt, 2020; Lupi, Martinotti, & Di Giannantonio, 2017), although prevalence rates are not yet established.

Negative consequences that arise from increased alcohol intake and binge‐drinking are commonly reported, in particular, the effect on memory, cognitive function, and perception (NHS, 2019; Welch, 2017). Alcohol is absorbed into the bloodstream through the stomach lining (Hahn, Norberg, & Jones, 1997), thus reduced caloric intake before drinking would inevitably exacerbate these effects, increasing the body’s vulnerability to the consequences of Drunkorexia. With this considered, it is not surprising that research has shown students who engage in Drunkorexia behaviours to be at a higher risk of experiencing negative alcohol‐related consequences compared to non‐Drunkorexia groups (Tuazon et al., 2019). A link between engagement in Drunkorexia behaviours and binge‐drinking (consuming a large amount of alcohol in a short space of time; NHS, 2019) has also been made (Knight et al., 2017). It has been found that individuals who engage in Drunkorexia behaviours also engage in higher levels of binge‐drinking (which further exacerbates negative consequences, alcohol toxicity and health threats). In addition, consistent and regular engagement in Drunkorexia behaviours has been found to increase the likelihood of developing an eating disorder or substance use disorder (Roosen & Mills, 2015).

While Drunkorexia is not currently a classified disorder, Rahal, Bryant, Darkes, Menzel, and Thompson (2012a, 2012b), developed the Compensatory Eating and Behaviours in Response to Alcohol Consumption scale (CEBRACs), which is frequently used in research to show the extent and prevalence of the behaviours (Bryant et al., 2012; Hill & Lego, 2019). The debate as to whether Drunkorexia is an eating disorder or substance abuse disorder is still ongoing, with evidence suggesting it cannot solely be classified as either (Griffin & Vogt, 2020; Hill & Lego, 2019; Hunt & Forbush, 2016). As a result, Choquette, Rancourt, and Kevin Thompson (2018) suggested Drunkorexia be classified as Food and Alcohol Disturbance (FAD), rather than an eating disorder due to the complicated nature of these behaviours. By adapting Fairburn’s (2008) transdiagnostic eating disorder model, Choquette et al. (2018) suggested that individuals who over‐evaluate their weight and shape, may be at risk of engaging in more compensatory behaviours during alcohol use to eliminate negative feelings towards their body. While this would account for those that carry out these behaviours due to body dissatisfaction, motivators associated with alcohol misuse would not be accounted for. As a result of this, the nomenclature FAD is now widely used when referring to Drunkorexia behaviours.

To further understand predictors of Drunkorexia behaviours (as measured by the CEBRACs), several quantitative studies have been conducted. Predictors of engagement in Drunkorexia include disordered eating in Italian college students (Pietrabissa et al., 2018), high sensation‐seeking and poor body esteem in an American college sample (Hill & Lego, 2019), emotional dysregulation in a sample of young adults (Laghi et al., 2019), low appearance esteem and high levels of disinhibition in a mixed student, non‐student and previous student sample (Griffin & Vogt, 2020) and to avoid weight gain in both students and non‐students (Lupi et al., 2017; Roosen & Mills, 2015; Ward & Galante, 2015).

To date, the literature on Drunkorexia is almost exclusively quantitative in nature. While there are some qualitative literature studies that investigate aspects of Drunkorexia, such as the relationships between food and alcohol consumption in young adults in the United Kingdom (Scott et al., 2020), between physical activity and binge‐drinking in US college students (Dinger et al., 2018) or even the fear of weight gain and alcohol use (Peralta, 2002), no qualitative research has investigated all aspects of Drunkorexia collectively in one sample.

As such, the current study seeks to extend knowledge on Drunkorexia, and aims to qualitatively explore the reasons and motivations for compensatory behaviours in response to alcohol consumption (restricting calorie intake, purging, and excessive exercise) specifically before, during, and after alcohol consumption. To achieve this, qualitative interviews were conducted with a sample of 18–26‐year‐olds. This age range was chosen as it was reflective of the age ranges used in comparable quantitative studies (Griffin & Vogt, 2020; Lupi et al., 2017). A diverse sample, inclusive of both students, non‐students and previous students was sought, to also extend knowledge on potential differences in reasonings and motivators.

## Methods

### Design

A qualitative study design with semi‐structured interviews was used. This was deemed the most appropriate study design, due to semi‐structured interviews allowing for flexibility within interviews. The main questions of the interviews focused on food and eating habits in response to alcohol consumption, any dietary changes and the reasons for these, as well as the participants’ perceptions of the relationship between food and alcohol. The interview schedule was loosely based on the CEBRACS.

### Ethical considerations

The study was developed in accordance with the British Psychological Society (Code of Human Research Ethics, 2012)’s ethical guidelines, and approved by the University of Huddersfield’s Psychology ethics committee.

### Participants

The inclusion criteria for participants were as follows, participants 1) had to be aged 18–26 years, 2) must – at least – occasionally drink alcohol, 3) must have, at least on occasion, made adjustment to lifestyle habits in response to alcohol consumption, and 4) must not have a prior diagnosis of an eating disorder (to minimize vulnerability to any physical harm or psychological distress). An equal number of students, non‐students and previous students was aimed for; recruitment was continued until data saturation was reached at 10 participants (data saturation being defined as no new codes were needed during analysis). The characteristics of participants are reported in Table 1. No participants were excluded.

**Table 1:** Overview of participant demographics

Participant pseudonym	Occupation status	Age	Gender
Anna	Student	25	Female
Bella	Student	23	Female
Charlotte	Student	23	Female
Dan	Non‐student	21	Male
Emily	Non‐student	25	Female
Frances	Non‐student	24	Female
Gary	Previous student	25	Male
Lucy	Non‐student	21	Female
India	Non‐student	20	Female
Josie	Previous student	25	Female

### Procedure

After ethical approval, advertisements for this study were shared on the university’s research participation system (for course credits) and social media. The advertisement said that participants were sought for a study investigating decision‐making in regard to alcohol consumption, and was recruiting individuals who, at least on occasion, made adjustment to lifestyle habits in response to alcohol consumption. If interested, participants were advised to email the researcher (MH), to arrange a suitable date and time for interview. Prior to participation, all participants were emailed an information sheet and consent form, which they were asked to return before participation. All interviews were conducted via Microsoft Teams. Once the interview was terminated, participants were emailed a debrief sheet and were given the opportunity to ask any further questions. Interviews lasted approximately 20–30 min; all interviews were conducted by MH. Participants were given up to seven days post‐interview to withdraw. After passing of the 7‐day withdrawal period, Interviews were transcribed verbatim and analysed in NVivo (Version 12). No incentives or rewards were offered for participation, other than course credits if participants were students at the university (Table 2).

**Table 2:** Thematic table

Theme	Subtheme	Quotations
Theme 1: Appearance concerns as a motivator for Drunkorexia	1.1: Compensatory behaviours to combat appearance concerns	‘I wouldn’t want to be too bloated; I want to look nice in what I'm wearing’‐Charlotte (student) ‘You look nice, you feel more confident, you just look nicer overall, no wants to feel and look like a slob when they’re going out’ – Gary (previous student) ‘I know it might sound stupid coming from a guy, but I hate feeling bloated. I worry about my body image all the time you know. I want to enjoy my night and not feel self‐conscious’. – Dan (non‐student)
	1.2: Societal expectations	‘I would just go get a pump on the biceps, just to feel nice and look nice’‐Gary (previous student) ‘There is a pressure… an expectation to be slim and look nice so it makes you want to consume fewer calories it makes your image look better really, it seems to get worse the more popular Instagram and that get, as all the girls on there look amazing on nights out’. –Josie (previous student) ‘Most of the time when I’m out I’ll look at other girls in their outfits and admire their figures. I just want to feel comfortable, so I enjoy myself and feel good in my body I do not want to feel like a fat slob’ – Anna (student)
	1.3: Beverage choice	‘I usually drink gin as It makes me feel less bloated and full – Emily (non‐student) ‘I always stick to gin and vodka cause like they’re the lowest on slimming world’ – Josie (previous student) ‘… because whiskey doesn’t bloat me’ – Dan (non‐student)
	1.4: Occasion type and company	‘I'm always out, I don't want when I see people for them to be like 'oh god she has put on a lot of weight since I last saw her’ – Lucy (non‐student) ‘It differs from the company you’re going to be with as well. If I am going out with my friends, it does not bother me how I am looking or how I am feeling’ – Gary (previous student) ‘If I was having drinks with the girls as a girl's night in, we would usually have a pizza or something, and id usually be in my baggy loungewear so I'm not as bothered about what I look like because I can hide it’ – Anna (student)
	1.5: External appearance prioritized over internal well‐being	‘I suffer really bad with my stomach, like a lot of stomach issues and it flares up when I drink so I would make myself sick most of the time to help this’ – Frances (non‐student) ‘If I'm feeling sicky on the night and I cannot shift that feeling and cannot be sick ill make myself throw up. I suffer from bad acid reflux, which bloats my stomach more when I drink so I take my Rennies to help it’. – India (non‐student) ‘I will throw it all up in someone's back garden and not mention it to anyone, I do it to make me feel a bit better and to give my stomach a bit of room for more alcohol’. – Dan (non‐student)
	1.6: From appearance esteem to calorific food intake	‘I always think that when I'm drinking and I'm drunk that I'm not adding the calories like I think I won't get fat I just don't think about it because I'm so drunk’. Anna (student) ‘I’ll have plenty of it [food] … I’ll eat whatever when I'm drunk and wouldn't be bothered. I'm not as body conscious when I'm drunk so I'm nothing thinking like what calories I'm consuming because I have lost that thought process’ – Josie (previous student) ‘I’ll just crave fast food, I think it's just because I'm so drunk at the time, the last thing I'm going to think about is calories’ – Bella (student) ‘By the end of the night id eat anything, I would eat fast food, I don't care about calories that I'm consuming’ – Josie (previous student) ‘I’d say I'm more swayed to fast food after drinking, I don't think about what I am eating as much… even though I’ve concentrated on calories in the drinks, it’s like it all goes out of the window by the end of the night…’ – Frances (non‐student) ‘You would want something greasy’ – Gary (previous student)
Theme 2: Drunkorexia behaviours to get value for money	N/A	‘If I'm out and drinking and I want to stay at the level of drunkenness that I'm at then I will not eat’ – Josie (previous student) ‘If you’re planning on drinking loads alcohol and then the more eat you as well the alcohol doesn’t affect you as much. It obviously doesn’t get you drunk as much’ – Dan (non‐student) I will not eat before I go out so I can get more steaming, I also do not eat while I'm out, takes up room in my stomach for more alcohol’. – Dan (non‐student) ‘I mainly do it so I can get more drunk, so yes avoiding food when I'm out, helps me to get more drunk’ – Bella (student) ‘The thing is as well if I eat when I'm out I don't get drunk which is just a waste of money, can't be having that’ – Dan (non‐student) ‘You do not want to drink all day and then eat food during the night when you're out cause your soaking up all the alcohol so all that drink you've drunk and all that money you've just spent it just goes to waste’. – Gary (previous student) ‘I wouldn't eat while I'm out because when I'm out and I eat I just cannot get drunk at all and it takes me a lot and when I cannot get drunk, I feel like it is wasting my money’. – Anna (student)
Theme 3: “It’s just a pattern… something I’ve always done”*: Drunkorexia as a routine*	N/A	‘I'd do it every time I'm drinking, just routine and that’ – Gary (previous student) ‘It’s just a subconscious thing… you just automatically think like I cannot feel this way or look this way, it’s just a pattern… and something I’ve always done’ – Dan (non‐student) ‘I’d say it is every time I drink alcohol because it is just routine’ – Bella (student) ‘It will be the start of the week and I’ll try and prepare myself for the week with more healthy food choices’ – Josie (previous student) I would usually start the week off good again and start afresh, I'll work out at the gym and eat well again. I know lockdown has stopped this but yeah I’d usually go out every weekend. It is my routine ha‐ha. I try to do it to balance out everything I ate and drank when I was drunk or hungover’. – Anna (student)

### Analytical methods

Braun and Clarke (2006, 2014)’s reflexive Thematic analysis (TA) was chosen to analyse the interviews. Alternative analyses methods, such as Content analysis (CA) and Interpretative phenomenological analysis (IPA) were considered (Elo & Kyngäs, 2008; Smith & Osborn, 2015). However, CA’s focus on frequency of codes and categories, rather than interpretation, made this method unsuitable. While IPA may have been a valid alternative, the researcher team decided against this method due to its emphasis on subjectivity and individual meaning attributed to experiences. As one of the aims of this study was to investigate potential differences, and similarities, between the student, non‐student, and previous student participants, TA was deemed the most appropriate method of analysis. Another key strength of TA is the flexibility for both descriptive and interpretive analysis (Braun & Clarke, 2006, 2014). An inductive, bottom‐up approach was used, with no pre‐set ideas or concepts.

Braun and Clarke (2006)’s six steps were followed to analyse the data; these steps were (1) familiarization, (2) initial code generation, (3) searching and generating themes, (4) reviewing themes, (5) defining and naming themes, and (6) writing the report. MH coded the interviews, with assistance from KV; MH and KV developed the themes together based on the coding, and all three authors (KV, MH, and BG) reviewed the themes in accordance with the data, defined and named the themes. All authors contributed to the writing of the report.

## Results

**Graph 1:**
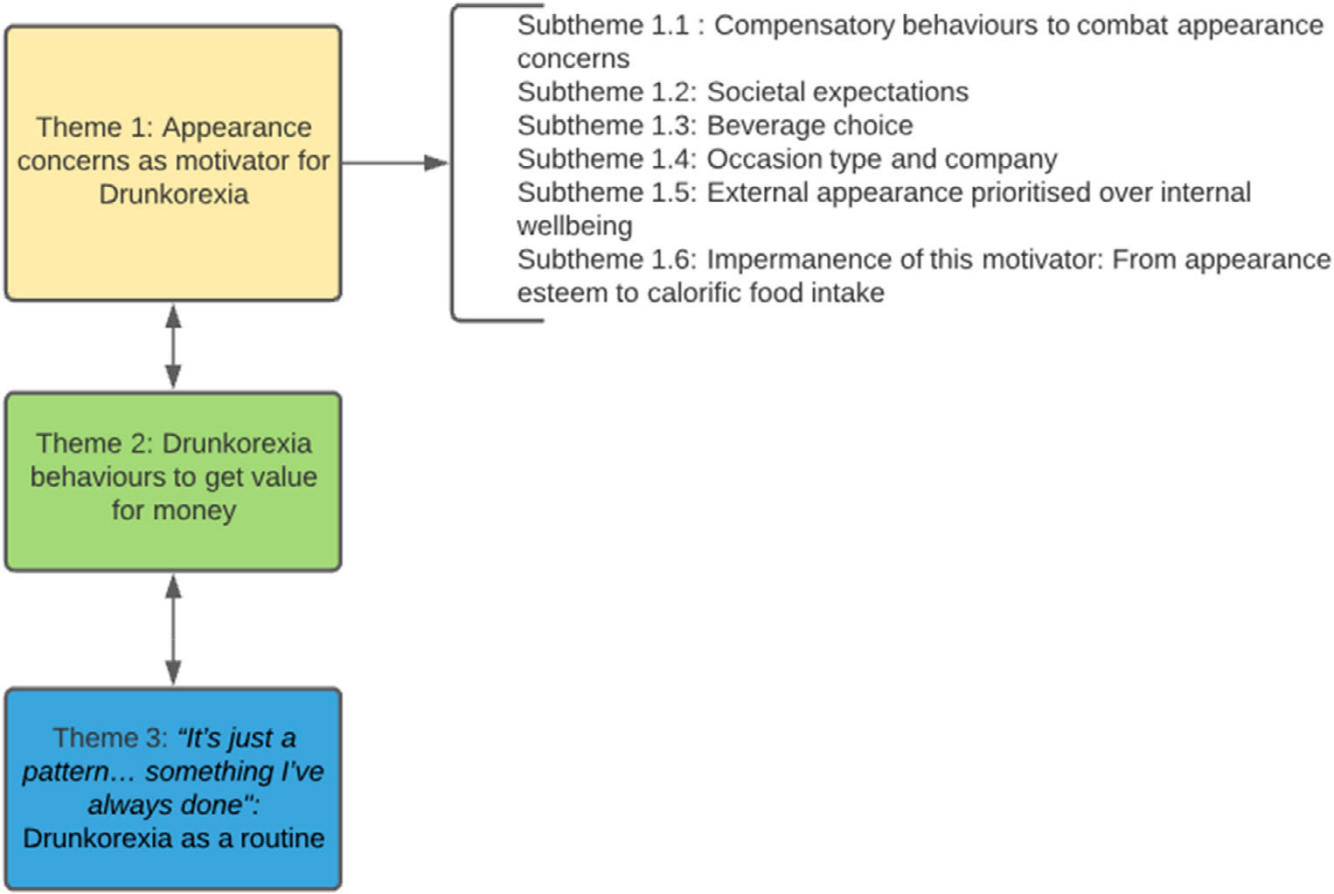
Graphic of themes.

Three themes were developed from the interviews, these were (1) Appearance concerns as a motivator for Drunkorexia, (2) Drunkorexia behaviours to get value for money, and (3) “It’s just a pattern… something I’ve always done”*: Drunkorexia as a routine*. Graph 1 provides an overview of the themes.

### Theme 1: Appearance concerns as a motivator for Drunkorexia

#### Compensatory behaviours to combat appearance concerns

In the interviews, it became obvious that appearance was a significant concern for participants, and that engagement in Drunkorexia behaviours served as a strategy to positively influence looks. Interestingly, both male and female participants reported engaging in these behaviours to avoid bloating on “nights out”, to look “skinny” (Anna, student), “good” (Charlotte, student) or “nice” (Gary, previous student), and to feel “comfier” in their clothes (Emily, non‐student).

“I would eat low‐calorie foods to keep myself feeling skinny before I go out… I just do not want to be bloated for my night out, so I tend to not eat.” Anna (student)

“You look nice, you feel more confident… no wants to feel and look like a slob when they’re going out.” ‐ Gary (previous student).

“I know it might sound stupid coming from a guy, but I hate feeling bloated. I worry about my body image all the time you know. I want to enjoy my night and not feel self‐conscious.” ‐ Dan (non‐student)

The following Drunkorexia behaviours were reported: restricting food intake, adjusting meal timing, consuming only low‐calorie foods, excessive exercising, and laxative use before alcohol consumption. It is interesting to note that the duration of engagement in these behaviours varied, with some participants reporting engaging in compensatory behaviours only on the day of planned alcohol consumption, while others stated adjustments to behaviours were made for up to a week before the drinking event (“would eat low‐calorie foods all week”, Bella, student). Both restrictive eating and laxative use was reported, to allow the participants “time to digest… food” (Bella, student)) or “to flush out” their bodies to make them “feel slimmer” (Josie, previous student), while Gary, who was a previous student, reported going to the gym to “pump the biceps” to look “feel nice and look nice”.

#### Societal expectation

Participants also drew a link between the use of compensatory behaviours and meeting the expectations of looking slim (for women) or muscular (for men) when out in public, as well as on social media photos. As such, it appeared that these restrictive or compensatory behaviours (reduced eating, increased exercising) were deemed ‘normal’ and necessary, in order to comply with the “pressure to look a certain way” (Josie, previous student). There was also an element of social comparison, where especially women, compared themselves to how others looked and dressed, wanting to look equally as good as others, both on nights out and on social media, and not like “a fat slob” (Anna, student). Anna (student) perceives feeling good in her body and being “comfortable” as the opposite to “feeling like a flat slob”.

“There is a pressure… an expectation to be slim and look nice so it makes you want to consume fewer calories it makes your image look better really, it seems to get worse the more popular Instagram and that get, as all the girls on there look amazing on nights out.” ‐ Josie (previous student).

“Most of the time when I’m out I’ll look at other girls in their outfits and admire their figures. I just want to feel comfortable, so I enjoy myself and feel good in my body I do not want to feel like a fat slob.” ‐ Anna (student).

What was also interesting was that one participant, who reported previously engaging in purging behaviours in response to alcohol consumption, reported never altering her food‐related behaviour as her “frequency of going out is so slim” that she is “not bothered about what” she is “eating and drinking because it is a treat to go out… so yes, not bothered or worried about any weight gain on that night” (Frances, non‐student). The use of purging for this individual was predominantly to alleviate problems with her stomach, as a result of drinking. It could be argued that this compensatory behaviour is in response to drinking alcohol per se, rather than in response to appearance concerns relating to alcohol. Furthermore, Frances stated that they rarely go out and so do not feel the need to change their eating behaviour, thus suggesting that Drunkorexia may be associated with heavier (and more frequent) drinking cultures rather than alcohol consumption per se. Reasons for this could be that individual with higher frequency of going out may be more worried about the calorific content of alcohol.

#### Beverage choice

Interestingly, concern about appearance were not just linked with engagement in compensatory behaviours, but also beverage choice by both women and men. By being aware of the calories within alcoholic beverages, and thus adjusting drink choice to low‐calorie drinks to avoid bloating, there is a danger of these participants experiencing negative alcohol‐related consequences as the drinks lower in calories that were mentioned were all stronger alcoholic drinks, such as whiskey, gin or vodka, and thus result in higher blood alcohol levels.

“I usually drink gin as It makes me feel less bloated and full.” ‐ Emily (non‐student).

“I always stick to gin and vodka cause like they’re the lowest on slimming world.” ‐ Josie (previous student)

“… because whiskey doesn’t bloat me.” ‐ Dan (non‐student).

#### Occasion type and company

In the interviews, both the type of planned drinking and the company it occurred with, was portrayed as decisive as to whether compensatory behaviours were engaged in or not, and discussed in relation to appearance concerns. Generally, ‘nights out’ in clubs or bars were reported as eliciting more compensatory behaviours than social drinking in the comfort of participants' homes. For example, Anna (student) reported that nights in with friends would not make her want to engage in compensatory behaviours, but going out drinking would.

“I'm always out, I don't want when I see people for them to be like 'oh god she has put on a lot of weight since I last saw her.” ‐ Lucy (non‐student)

Interestingly one male participant stated that when he would go out with friends, he would not “be bothered” how he was “looking or feeling” (Gary, previous student). Thus, there seem to be differences as to how the company for drinking influences engagement in Drunkorexia. Perhaps there are links to be drawn between group size, appearance esteem, and self‐consciousness, with participants feeling less need to engage in compensatory behaviours in small and more intimate gatherings (both with friends, and at home) compared to nights out (with larger crowds, including friends, acquaintances, and strangers).

“It differs from the company you’re going to be with as well. If I am going out with my friends, it does not bother me how I am looking or how I am feeling.” ‐ Gary (previous student)

The type of planned drinking as well as company for the drinking were also directly linked with outfit and food choice, such as “pizza” and “baggy loungewear” (“to hide bloating” – Anna, student) for nights in with familiar people and the opposite for nights out in clubs or bars. For these instances, no concerns about calorie intake were listed, but the bloating was compensated for by clothing choice.

#### Drunkorexia engagement despite negative consequences

So far it appears as though appearance‐related concerns (Theme 1) are driving motivators for engagement in Drunkorexia behaviours, and that these behaviours aid participants in feeling good about themselves. At the same time however, participants also report negative consequences of compensatory behaviours, including feeling sick after drinking large amounts on (largely) empty stomachs or acid reflux. As a consequence, some participants would self‐induce vomiting, then continue drinking afterwards or to “make room for more alcohol” (Dan, non‐student). This suggests that participants are ascribing a higher priority to looking good externally than feeling good internally. One participant, India (non‐student), even reported medicating herself with a dual purpose: to medicate her acid reflux, which is exacerbated when drinking, and to reduce bloating. By being prepared with heartburn tablets for this issue, the participant is aware of how drinking alcohol will affect her health, but enjoying her night drinking is more important to her. India also reported self‐inducing vomiting when feeling unwell as a result of alcohol. Another participant also reports self‐inducing vomiting, and hints at the unspoken nature of compensatory behaviours, by saying he would make himself “sick” away from “anyone” (Dan, non‐student), which may suggest that these behaviours are either stigmatized, he feels as though he may be judged for it or may allude to the fact that he does not want to be observed being vulnerable and throwing up.

“If I'm feeling sicky on the night and I cannot shift that feeling and cannot be sick ill make myself throw up. I suffer from bad acid reflux, which bloats my stomach more when I drink so I take my Rennies to help it.” ‐ India (non‐student)

This suggests that participants place a higher value in engaging in compensatory behaviours in response to alcohol consumption to achieve their desired looks and appearance, than their well‐being, despite being aware of the negative consequences of alcohol consumption (such as exacerbating stomach issues). As with the other themes, differences between the occupational groups were not discernible, suggesting that consequences are not deterrent enough, to avoid Drunkorexia engagement.

#### From appearance concerns to calorific food intake

Most interestingly, all participants who reported restricting food intake before alcohol consumption, also reported eating very unhealthy, greasy, and calorific foods at the end of nights out before going home. This suggests that the need to restrict food is then either absent once drunk (“the last thing I'm going to think about is calories” ‐ Bella, student) or the occasion which triggered compensatory behaviours was deemed as over. These food choices were even reported by those participants who described being concerned with their appearance, with one participant suggesting that their initial ‘thought process’ (Josie, previous student) was ‘lost’ during/after drinking; this may potentially hint at increased disinhibition with increased alcohol intake. This new insight into Drunkorexia is novel, and allows deeper understanding of the nature and classification of Drunkorexia.

“I always think that when I'm drinking and I'm drunk that I'm not adding the calories like I think I won't get fat I just don't think about it because I'm so drunk.” ‐ Anna (student)

“… I’ll eat whatever when I'm drunk and wouldn't be bothered. I'm not as body conscious when I'm drunk so I'm nothing thinking like what calories I'm consuming because I have lost that thought process.” ‐ Josie (previous student).

“… because I'm so drunk at the time, the last thing I'm going to think about is calories.” ‐ Bella (student)

### Theme 2: Drunkorexia behaviours to get value for money

In addition to engaging in Drunkorexia‐behaviours as a result of appearance‐related concerns, participants also reported doing so to achieve both quicker, and heavier, intoxication.

“If you’re planning on drinking loads alcohol and then the more eat you as well the alcohol doesn’t affect you as much. It obviously doesn’t get you drunk as much… I will not eat before I go out so I can get more steaming, I also do not eat while I'm out, takes up room in my stomach for more alcohol.” ‐ Dan (non‐student).

“I mainly do it so I can get more drunk, so yes avoiding food when I'm out, helps me to get more drunk.” ‐ Bella (student)

Being a student is often associated with having less money to spend, therefore, the explanation for students’ engagement in Drunkorexia behaviours as an economical choice can be made. However, this reasoning was reported across all participant groups (students, non‐students, and previous students), and therefore may not be valid and cannot account for the same reasoning across occupation groups.

“The thing is as well if I eat when I'm out I don't get drunk which is just a waste of money, can't be having that.” ‐ Dan (non‐student)

“You do not want to drink all day and then eat food during the night when you're out cause your soaking up all the alcohol so all that drink you've drunk and all that money you've just spent it just goes to waste.” ‐ Gary (previous student)

“I wouldn't eat while I'm out because when I'm out and I eat I just cannot get drunk at all and it takes me a lot and when I cannot get drunk, I feel like it is wasting my money.” ‐ Anna (student)

Interestingly, two of the participants who proposed ‘value for money’ as their main motivator for engagement in compensatory behaviours, also reported that they would often go out shopping for outfits before going out. [Charlotte ‐ student; Gary ‐ previous student]. Therefore, money concerns per se may not be the prime motivator for wanting ‘value for money’ and may explain why this reasoning was reported across groups.

Perhaps, this is an inclination to the idea that clothes are tangible, more permanent physical investments, whereas the consumption of alcohol is non‐tangible and temporary. Or, this reasoning may be directly linked to appearance‐related concerns, where buying new clothes to look ‘good’ would be the motivator for this spending.

However, it must also be noted that the idea of ‘value for money’ is somewhat contradicted by participant Dan (non‐student) who reported both wanting value for his money but also self‐inducing vomiting on nights out, to make more ‘room’ for alcohol.

### Theme 3. ‘It’s just a pattern… something I’ve always done’: Drunkorexia as ‘a routine’

It is important to understand the frequency with which participants engage in Drunkorexia behaviours. In the interview, all but one participant (Frances) reported engaging in binge‐drinking on a weekly basis and can thus be defined as heavier binge‐drinkers. Furthermore, eight of the 10 participants explained that engagement in compensatory behaviours prior to alcohol consumption was part of their ‘going out’ routine. For these participants, compensatory behaviours appeared to go hand in hand with alcohol consumption, and Drunkorexia is portrayed as normalized and part of the routine for events where heavier drinking is involved. This also links with Theme 1, and the duration in which these compensatory behaviours are engaged in (ranging from hour to days before drinking). Interestingly, in the interviews, there were no differences identifiable between women and men, nor students, previous students, and non‐students; suggesting Drunkorexia is prevalent within all three occupational groups and in both genders included in the study.

“I'd do it every time… just routine and that.” ‐ Gary (previous student)

“It’s just a subconscious thing… you just automatically think… it’s just a pattern… and something I’ve always done.” ‐ Dan (non‐student)

“I’d say it is every time I drink alcohol because it is just routine.” ‐ Bella (student)

“… I’d usually go out every weekend. It is my routine… to balance out everything I ate and drank when I was drunk or hungover.” ‐ Anna (student)

## Discussion

This study sought to extend the literature on Drunkorexia, by exploring underlying reasons, thought processes, and motivations for the use of compensatory behaviours (restricting calorie intake, purging, and excessive exercise) in response to alcohol consumption in a sample of 18–26‐year‐olds. Analysing the collected semi‐structured interviews with Thematic Analysis (Braun & Clarke, 2006) led to the development of the following three themes: 1) Appearance concerns as a motivator for Drunkorexia, 2) Drunkorexia behaviours to get value for money and 3) “It’s just a pattern… something I’ve always done”: Drunkorexia as a routine. In detail, participants reported wanting to look better, slimmer, and more comfortable in their clothes when out drinking and emphasized that both the company and the type of event in which drinking occurred would influence the likelihood of Drunkorexia behaviours occurring. In addition, participants described wanting value for their money, and that Drunkorexia behaviours were part of their routine and automatic behaviours. While previous qualitative studies have investigated the relationship between alcohol consumption and some compensatory behaviours (Dinger et al., 2018; Giles & Brennan, 2014; Peralta, 2002; Scott et al., 2020), this study is the first to explicitly explore Drunkorexia, the timing of compensatory behaviours, and underlying motivators qualitatively.

Thus, the results expand the knowledge of reasons for engagement in Drunkorexia behaviours, with appearance concerns being the most prominent reason for engagement in Drunkorexia behaviours pre‐alcohol consumption. This finding is in accordance with past quantitative research: it supports Griffin and Vogt (2020)’s finding that appearance esteem is a significant predictor for Drunkorexia engagement. Previously, weight esteem was also found to be significant predictor of Drunkorexia engagement (Lupi et al., 2017), however the current results do not support this. While participants did describe wanting to be appear slimmer, the consumption of high‐calorie and greasy food reported at the end of drinking events does not support weight esteem as a predictor for Drunkorexia behaviours. Participants clearly drew a link between appearance (i.e., not wanting to look “like a fat slob”) and Drunkorexia behaviours, rather than between weight and Drunkorexia.

However, this study also suggests that the picture is more complex, by highlighting that these compensatory behaviours and the initial motivators do not persist. Instead, at the end of the drinking occasion, participants reported a disregard for compensatory behaviours and appearance esteem. This has previously not been reported. It is also interesting that participants never discussed engaging in compensatory behaviours prior to alcohol consumption to compensate for the calories consumed in takeaway food at the end of the night, and only ever mentioned concern regarding the calories in alcohol as a motivator for compensatory behaviours.

These are novel findings and require further exploration.

Firstly, appearance concerns were often in relation to social media and pictures being posted online from drinking occasions. Participants described wanting to look good on social media posts or comparing themselves to others on social media; which is coherent with past research (Atkinson & Sumnall, 2016; Choukas‐Bradley, Nesi, Widman, & Higgins, 2018; Zheng, Ni, & Luo, 2019). Social media posts and uploads about events involving alcohol consumption have increased significantly over the last decade (Hendriks, van den Putte, Gebhardt, & Moreno, 2018; Moreno & Withehill, 2014; Phan, Muralidhar, & Gatica‐Perez, 2019). A recent cross‐cultural study also reported that, in their large sample of adolescents and young adults (15–25 years old, from the United States, Spain, South Korea, and Finland), uploading pictures to social media (via Facebook and Instagram) may be a potential facilitator for social media‐related hazardous alcohol consumption (Savolainen et al., 2020). This may directly reflect the results of the current study: participants (both males and females) wanted to look “good” or “slim” and “not bloated” on social media posts, which they hoped to achieve via engagement in Drunkorexia behaviours (i.e., hazardous alcohol consumption), but are then even more likely to suffer negative alcohol‐related consequences, as reported by participants in the study and the wider literature.

Secondly, the current results further support the argument that Drunkorexia should not be classed as either an eating or substance use disorder, but as a FAD. In fact, the current findings reflect Choquette et al. (2018)’s suggestion that individuals who over‐evaluate their shape, may be at risk of engaging in more compensatory behaviours during alcohol use to eliminate negative feelings towards their body. This study did not directly support the over‐evaluation of weight as a predictor for Drunkorexia engagement, as also suggested by Choquette et al. (2018), the relationship between appearance and weight may not be linear.

In addition, while participants suggested that their Drunkorexia behaviours were a routine in response to alcohol consumption, Drunkorexia behaviours were always displayed in response to a drinking occasion and dependent on a number of factors, such as company and type of event, rather than a persistent pattern of maladaptive behaviour that occurs at all times as, for example, in Bulimia or Anorexia Nervosa. Therefore, this strengthens the argument that Drunkorexia should be classed as a FAD, rather than a disorder. It also cannot be classed as a substance use disorder, as due to the event‐ and company‐related aspects, there is no persistent use of the substance, as one would expect to see in a substance use disorder.

In contrast, engagement in Drunkorexia appears to be the result of a rational decision‐making progress made by participants before drinking alcohol, therefore, the current findings also open the discussion whether Drunkorexia truly is a FAD, or a lifestyle behavioural choice. The third theme ‘Drunkorexia behaviours to get value for money’ reveals further information about predictors and motivators for Drunkorexia. In the crudest sense, getting ‘value for money’ from purchased alcohol means experiencing the maximum intoxication effect of alcohol on the body. This links with previous findings by Griffin and Vogt (2020) as well as Hill and Lego (2019) who reported sensation seeking, particularly disinhibition, as predictors for Drunkorexia engagement. Feeling the maximum intoxication effect of (purchased) alcohol may indeed link with sensation seeking. Viewed as a psychobiological personality trait, sensation‐seeking incorporates an individual’s desire for novelty, intensity, and complexity in their experiences (Arnett, 1994; Roberti, 2004). High sensation‐seeking is a well‐known predictor for increased alcohol use and hazardous drinking (Hirvelä, Sipilä, & Keski‐Rahkonen, 2022; Hittner & Swickert, 2006; Lac & Donaldson, 2021; Oberlin et al., 2021; Sznitman & Engel‐Yeger, 2017; Yanovitzky, 2006). Thus, it could be suggested that the ‘value for money' mentality is employed to achieve/experience the maximum sensation of intoxication effects. Furthermore, the disinhibition factor of sensation‐seeking refers to engagement in less acceptable forms of sensation‐seeking, including hazardous drinking and poor‐decision making (Carlson, Johnson, & Jacobs, 2010; Colby, Colby, & Raymond, 2009; Orlebeke, Van Der Molen, Dolan, & Stoffels, 1990); which may explain increased engagement in Drunkorexia in individuals with high disinhibition traits. However, this cannot explain the whole picture as although increasing intoxication (i.e., feeling the full effects of alcohol) was discussed by participants as a motivator for Drunkorexia, the responses still returned to appearance, as one female participant stated that she was no longer body conscious when intoxicated. Thus alcohol consumption may be used to mask any self‐doubt or body‐conscious thoughts. This was also argued by Hill and Lego (2019) who suggested poor appearance esteem as a predictor for Drunkorexia may be due to self‐worth‐related variables.

Another important finding is that, in the current sample, all participant groups reported similar reasons for engagement in Drunkorexia behaviours. This also further strengthens the argument against Drunkorexia as a student lifestyle choice, such as made by Griffin and Vogt (2020). Perhaps, the findings reflect a more consumerist, economical decision‐making process, to gain value for money, regardless of income, or perhaps that all participants wanted to ‘look good’ on nights out drinking due to societal expectations, pressure they put on themselves and social media.

### Limitations

There are several limitations to the current study.

Firstly, there are limitations regarding sample size: there are only 10 participants. However, the small, purposive sample allowed the researchers to conduct in‐depth analyses of participants’ accounts and present a “new and richly textured understanding” (Sandelowski, 1995, p. 183) of FAD; thus meeting the sample requirements for qualitative research (Vasileiou, Barnett, Thorpe, & Young, 2018). Despite the argument that qualitative research does not allow for generalizability to other samples, or the population at hand, the results of this study are important and add value to the classification of Drunkorexia as FAD, especially in the context of previous research, as is one of the quality markers of qualitative research (Carminati, 2018).

Secondly, the sample also had an uneven split of genders. The implications of this uneven gender split are difficult to predict, as some studies have found differences in Drunkorexia behaviours between the male and female gender (Eisenberg & Fitz, 2014; Hunt & Forbush, 2016; Roosen & Mills, 2015) while others, such as Griffin and Vogt (2020), have not.

Thirdly, due to the study advertising for participants who, on occasion, alter their behaviour regarding alcohol consumption, participants may have experienced interview bias, in which they reported what they perceived the interviewer wanted to hear. However, as there were some differences as to when and why the behaviour was engaged in, it suggests that participants felt able to express themselves freely.

Fourth, the impact of COVID‐19 on the validity of the current findings must also be noted: while participants were readily able to report on their alcohol consumption and compensatory behaviours in response to alcohol, participants had to – some degree – rely on retrospection, which may have distorted their experiences. However, the authors are confident that the impact of this is minimal, due to the findings resonating with much of the published literature (pre COVID‐19 pandemic and restrictions).

### Clinical implications

This study supports the idea that Drunkorexia be viewed as a FAD, or perhaps even as a rational decision‐making process behind the behaviour, which may be to look good on nights out, to get value for money, experience higher intoxication effects, or a combination of these. The results also further show that Drunkorexia is prevalent across the age group of 18–26 year olds, regardless of occupation, and can have negative consequences on the individual. The current results (along with other recent research, such as Booker et al., 2021) thus emphasize that (mental) health practitioners should be aware of, and recognize, the different aspects of FAD (restricting calorie intake, purging, and excessive exercise), and to be able to explain risks, and sign‐post to appropriate resources.

### Recommendations for future research

There is much work to be done regarding FAD. Firstly, participants referred to Drunkorexia engagement being an automatic process and a routine; therefore, the automaticity of this process needs to be further established and a qualitative large‐scale study regarding decision‐making processes may yield further insight.

Secondly, further research needs to be conducted, especially in the United Kingdom, to fully understand the prevalence and predictors in larger, nationally representative samples, to assess how widespread engagement in FAD is. The research is still predominantly being conducted in the United States; although FAD has now been shown to also be prevalent in 18–26 year olds in the United Kingdom.

Thirdly, parallels can be drawn between binge‐drinking and Drunkorexia, and Drunkorexia may even overlap with binge‐drinking. There are a large number of interventions that have been developed to tackle binge‐drinking in the population of 18–26‐year‐olds, yet no intervention for Drunkorexia has yet been developed. Therefore, future research must seek to apply established behaviour change techniques which have previously been used to successfully reduce binge‐drinking, to develop interventions for Drunkorexia, such as self‐affirmation theory, intention implementations, such as ‘if… then’ statements, and the theory of planned behaviour (Armitage, Harris, & Arden, 2011; Armitage, Rowe, Arden, & Harris, 2014; Knight & Norman, 2016; Norman & Wrona‐Clarke, 2015; Norman et al., 2018; Vogt, Stephenson, & Norman, 2021). The authors argue that self‐affirmation in particular may prove effective in this regard (Steele, 1988). It would be important to investigate whether self‐affirmation in an unrelated, non‐appearance/weight‐related domain can reduce appearance‐esteem and/or weight‐esteem, both which have been found to predict Drunkorexia engagement (Griffin & Vogt, 2020; Hill & Lego, 2019). Thus, studies should assess appearance and weight esteem pre‐ and post‐intervention, as well as the behaviours of interests (alcohol consumption, frequency of binge‐drinking, frequency of bulimia behaviours, and frequency and intensity of physical activity).

### Conclusion

In conclusion, this study supports the view of Drunkorexia as a FAD, rather than a clinical disorder (neither eating nor substance use disorder). In this study, engagement in Drunkorexia appears to be predominantly appearance‐related, with social media and social comparisons playing significant roles. In addition, there appears to be a routine and habitual connotation. This study, in addition to other literature which has included larger samples (Giles & Brennan, 2014; Griffin & Vogt, 2020; Hill & Lego, 2019; Lupi et al., 2017; Roosen & Mills, 2015; Scott et al., 2020), emphasizes that the age group of 18–26‐year‐old are at risk of engagement in Drunkorexia and its negative health‐related consequences, regardless of occupation/student status, and that the need for interventions is pressing.

## Conflicts of interest

All authors declare no conflict of interest.

## Author contribution

**Katharina Sophie Vogt:** Conceptualization; Formal analysis; Investigation; Methodology; Project administration; Resources; Software; Supervision; Validation; Visualization; Writing – original draft; Writing – review & editing. **Michela Harper:** Investigation; Methodology; Writing – original draft. **Bethany Leigh Griffin:** Methodology; Project administration; Resources; Writing – review & editing.

## Data Availability

The data that support the findings of this study are available from the corresponding author upon reasonable request.
